# A New 1,4-Diazepine from South China Sea Marine Sponge *Callyspongia *Species

**DOI:** 10.3390/molecules15020871

**Published:** 2010-02-10

**Authors:** Ri-Ming Huang, Wei Ma, Jun-De Dong, Xue-Feng Zhou, Tunhai Xu, Kyung Jin Lee, Xianwen Yang, Shi-Hai Xu, Yonghong Liu

**Affiliations:** 1 Key Laboratory of Marine Bio-resources Sustainable Utilization, South China Sea Institute of Oceanology, Chinese Academy of Sciences, Guangzhou 510301, China; E-Mail: huang_riming@hotmail.com (R.M.H.); 2 Graduate University of the Chinese Academy of Sciences, Beijing 100049, China; 3 Guangzhou First Municipal People’s Hospital, Guangzhou 510180, China; 4 National Experiment Station of Tropical Marine Biology, Sanya 572000, China; 5 School of Chinese Materia Medica, Beijing University of Chinese Medicine, Beijing 100102, China; 6 Invertebrate Research Division, National Institute of Biological Resources, Environmental Research Complex, Incheon 404170, Korea; 7 Department of Chemistry, Jinan University, Guangzhou 510632, China

**Keywords:** marine sponge, *Callyspongia* sp., diazepine, callysponine

## Abstract

A new 1,4-diazepine, callysponine (**1**), was isolated from a South China Sea *Callyspongia *sp. marine sponge, together with four known proline-based diketopiperazines: cyclo-(*S*-Pro-*R*-Leu) (**2**), cyclo-(*S*-Pro-*R*-Val) (**3**), cyclo-(*S*-Pro-*R*-Ala) (**4**), and cyclo-(*S*-Pro-*R*-Tyr) (**5**). The new structure was determined on the basis of NMR and MS analysis, and the absolute stereochemistry was defined by NOESY spectroscopy and optical rotation. The structures of the known compounds were identified by comparison of their spectroscopic data with those reported in the literature. Callysponine (**1**) did not inhibit the growth of HepG2 (hepatoma carcinoma cell), A549 (lung carcinoma cell), and HeLa (cervical cancer cell) cell lines.

## 1. Introduction

Marine sponges of the genus *Callyspongia* are known as common sources of structurally unique and biologically active natural products, such as polyacetylenes [1], peptides [2], terpenoids [3], alkaloids [4], fatty acids [5], polyketides [6], sterols [7], peroxides [8], and butenolides [9]. Some of these compounds possess antifouling [10], cytotoxic [11], anticancer [6], and antimicrobial [10] properties. 

In our study of bioactive compounds from *Callyspongia* sp. marine sponges collected from the coast of Hainan, the new 1,4-diazepine **1**, as well as proline-based diketopiperazines **2**–**5** were obtained. The structure of the new compound was elucidated by the aid of COSY, HSQC, HMBC, and MS experiments, while the absolute stereochemistry of **1** was defined by NOESY spectroscopy and optical rotation. Compounds **2**–**5** were identified as cyclo-(*S*-Pro-*R*-Leu) (**2**) [12], cyclo-(*S*-Pro-*R*-Val) (**3**) [12], cyclo-(*S*-Pro-*R*-Ala) (**4**) [13], and cyclo-(*S*-Pro-*R*-Tyr) (**5**) [14], respectively, by comparison of their spectroscopic data with those reported in the literature.

## 2. Results and Discussion

**Figure 1:**
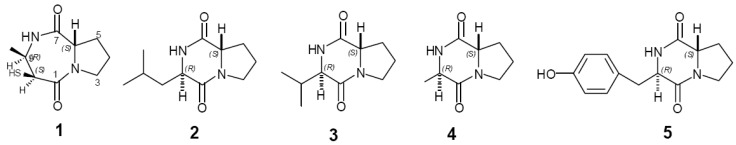
Structures of compounds **1**–**5**.

The aqueous EtOH extract of the *Callyspongia* sp. marine sponge was suspended in water, and partitioned successively with petroleum ether, EtOAc, and *n*-butanol. The EtOAc and *n*-butanol fractions were subjected to silica column, Sephadex LH-20, and ODS-HPLC, to yield compounds **1**–**5 **(Figure 1). 

**Figure 2:**
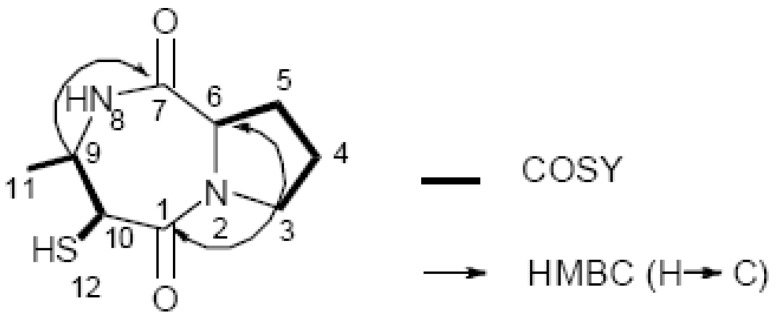
Key HMBC and COSY correlations of **1**.

Compound **1 **was obtained as yellow oil and had the molecular formula C_9_H_14_N_2_O_2_S, as deduced from the HREI mass spectrum (*m*/*z* 214.0774 [M]^+^; calc. for C_9_H_14_N_2_O_2_S, 214.0776) and NMR data (Table 1), the latter being unambiguously assigned by aid of COSY, HMQC, and HMBC experiments. ^1^H-NMR (Table 1) chemical shifts of two *α*-methine protons at *δ*_H_ 4.36 and 4.11, and ^13^C-NMR (Table 1) chemical shifts of two carbonyl carbons at *δ*_C_ 170.2 and 165.4, supported the presence of a peptide fragment. The fact that compound **1** was negative to the ninhydrin test, suggested a cyclic or a *N*-terminus-blocked peptide [15]. The ^1^H-NMR spectrum of **1** showed the presence of one methyl signal at *δ*_H_ 1.34 (d, *J* = 6.5 Hz, 3H), one thiol proton at *δ*_H_ 1.89 (m, 1H), three consecutive methylene signals (*δ*_H_ = 2.0–3.7), and three methine signals at *δ*_H_ 3.97 (br d, *J* = 3.0 Hz, 1H), 4.11 (t, *J* = 7.5 Hz, 1H), and 4.36 (dt, *J* = 6.5, 3.0 Hz, 1H), and one acid amide proton at *δ*_H_ 6.73 (s, 1H). The ^13^C-NMR spectrum of **1** showed the presence of two carbonyl carbons (*δ*_C_ 170.2 and 165.4), two *N*-methine carbons (*δ*_C_ 65.6 and 59.0), one *N*-methylene carbon (*δ*_C_ 45.3), one *S*-methine carbon (*δ*_C_ 59.4), and one methyl carbon (*δ*_C_ 18.9). The remaining carbons were assigned to two methylene carbons (*δ*_C_ 28.1 and 22.6). The COSY spectra (Figure 2) indicated three consecutive methylenes (*δ*_H_ 2.0–3.7) characteristic of a proline residue [16], and showed correlations of H-9 with H-10 and H-11. The assignments of a remaining C_3_H_6_S fragment, and one site of unsaturation were carried out by 2D NMR experiments. Key HMBC correlations H-9/C-7, H-3a/C-1, H-3a/C-6, H-3b/C-1, H-3b/C-6, H-11/C-9, and H-11/C-10, as well as COSY connectivities (Figure 2), were indicative of a pyrrolidine-fused seven-membered diazepine ring. The placement of a methyl group at C-9, and a thiol group at C-10, were deduced from the COSY correlations H-11/H-9, and SH-12/H-10, respectively.

**Table 1:** ^1^H- (500 MHz) and ^13^C-NMR (125 MHz) data of compound **1** (in CDCl_3_, *δ* in ppm, *J* in Hz).

No.	*δ*_c_	*δ*_H_	HMBC (H to C)
1	165.4	–	–
2	–	–	–
3	45.3	3.51 (m)	C-1, 4, −5, −6
		3.61 (m)	C-1, 4, −5, −6
4	22.6	2.00 (m)	C-3, −5, −6
		2.06 (m)	C-3, −5, −6
5	28.1	2.34 (m)	C-4, −6, −7
			C-4, −6, −7
6	59.0	4.11 (t, 7.5)	C-4, −5, −7
7	170.2	–	–
8	–	6.73 (s)	–
9	65.6	4.36 (dt, 6.5, 3.0)	C-7, −11
10	59.4	3.97 (d, 3.0)	C-1, −9, −11
11	18.9	1.34 (d, 6.5)	C-9, −10
12	–	1.89 (m)	–

**Figure 3:**
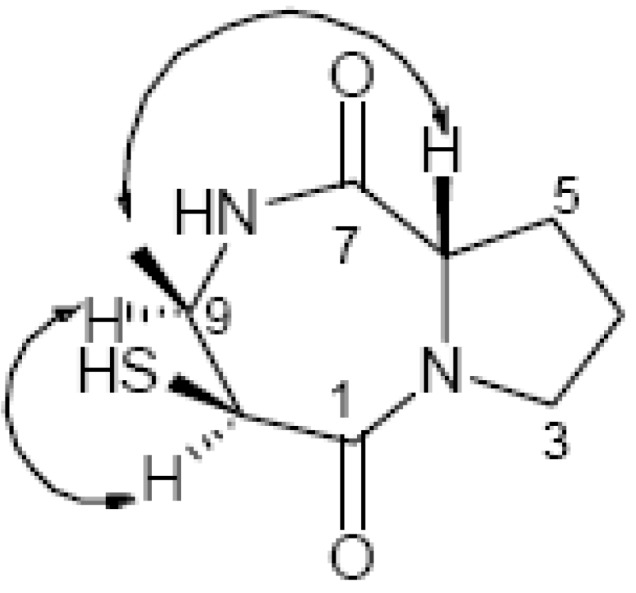
Key NOE correlations of compound **1**.

The absolute configuration of compound **1** was determined by the optical rotation, NOESY spectrum and analysis of the coupling constants (*J*), along with inspection of the molecular model. In the NOESY spectrum (Figure 3), the key NOE correlations of H-9/H-10 and H-6/H-11 showed that H-9 and H-10, H-6 and H-11 were on the same face, so the relative stereochemistry was determined. The coupling patterns of the H-9 (dt, *J* = 3.0 and 6.5 Hz) and H-10 (d, *J* = 3.0 Hz) led to confirmation of the *cis*-orientation of H-9/H-10 and possessed *β*-orientation [17], and thus the configuration of C-9 and C-10 were determined as R* and S*, respectively. The sign of [*α*]_D_ for proline-containing DKPs is either negative or positive, depending only on the absolute configuration of Pro [12]. On the basis of the sign of [*α*]^20^_D_ (−52.6) and by comparison of the NMR data of the proline residue with those of proline-containing DKPs [12,13,14], which suggested C-6 has (*S*)-configuration, Pro in **1** has therefore (*S*)-configuration, and the above data approved the absolute configuration of compound **1** as the new (6*S*, 9*R*, 10*S*)-1,4-diazepine.

The structures of known compounds **2**–**5 **were confirmed by detailed NMR data comparison with those in the literature [12,13,14]. The absolute configuration of **2**–**5** were determined by comparison with reported optical rotation values [12,13,14].

Compound **1** was evaluated for cytotoxicity by the 3-(4,5-dimethylthiazol-2-yl)-2,5-diphenyltetrazolium bromide (MTT) method [18], and showed a marginal activity against a small panel of three human tumour HepG2 (hepatoma carcinoma cell), A549 (lung carcinoma cell), and HeLa (cervical cancer cell) cell lines, their inhibition ratio were lower than 10% at concentration of 100 μg/mL with the IC_50_ values of the positive control compound 5-Fu 13.70, 2.13, and 3.83 μg/mL, respectively.

## 3. Experimental

### 3.1. General

NMR spectra were recorded on a Bruker AC 500 NMR spectrometer with TMS as an internal standard. ESI-MS data were measured on a Agilent 1200 LC-MS spectrometer. HREIMS data were obtained from MAT 95XP (Thermo) mass spectrometer. The silica gel GF_254_ plates used for TLC were supplied by the Qingdao Marine Chemical Factory (Qingdao, China). Analytical HPLC was performed on a Hitachi L-2400 HPLC system, using a YMC ODS-H80 column (250 × 4.6 mm i.d., 4 μm) coupled to an Alltech ELSD 800 detector; semi-preparative HPLC was performed on a Hitachi L-2400 HPLC system, using a YMC ODS-H80 column (250 × 10 mm i.d., 4 μm) coupled to an Alltech ELSD 800 detector with flow-splitter valve (Parker: NS) set at a split ratio of 20:1 (collector: detector). Optical rotation data were measured by Perkin-Elmer Model 341 polarimeter. Spots were detected on TLC under UV light or by heating after spraying with 5% H_2_SO_4_ in EtOH (v/v).

### 3.2. Animal Material

The sponge is an undescribed species of *Callyspongia * (order Haplosclerida, family Callyspongiidae) collected off the coast of Hainan Island, South China Sea, in January, 2007. The specimen was identified by Dr. Kyung Jin Lee. A voucher specimen (No. 20070101) was deposited in Natural History Museum, Hannam University, Daejon, Korea and Key Laboratory of Marine Bio-resources Sustainable Utilization, South China Sea Institute of Oceanology, Chinese Academy of Sciences, China.

### 3.3. Extraction and Isolation

The wet sponges (10 kg) were extracted three times with EtOH/H_2_O (90:10, 20 L). The aqueous EtOH extract was concentrated under vacuum. The combined extract was partitioned between EtOAc and H_2_O. The EtOAc soluble portion (28 g) was partitioned between petroleum ether and EtOH/H_2_O (7:3, 500 mL). The EtOH/H_2_O soluble portion (8.0 g) was chromatographed on a silica gel column (80 g) eluted with petroleum ether (500 mL, fraction A), petroleum ether/EtOAc (8:2, 500 mL, fraction B), petroleum ether/EtOAc (1:1, 500 mL, fraction C), petroleum ether/EtOAc (3:7, 500 mL, fraction D), petroleum ether/ EtOAc (1:9, 500 mL, fraction E), EtOAc (500 mL, fraction F), EtOAc/acetone (1:1, 500 mL, fraction G), and MeOH (500 mL, fraction H), to give eight fractions. Fraction D (1.06 g) was subjected to column chromatography (CC) with gradient EtOAc/acetone (10:0, 250 mL; 8:2, 250 mL; 7:3, 250 mL; 5:5, 250 mL) to give four subfractions (D1–D4). Fraction D1 (45.2 mg) was purified by Sephadex LH-20 (CHCl_3_/MeOH, 2:8, 250 mL) to afford **3 **(13.3 mg). Fraction D4 (90.1 mg) was purified by Sephadex LH-20 (CHCl_3_/MeOH, 2:8, 250 mL) to yield **2 **(23.4 mg). Fraction G3-2 (112.3 mg) was purified by Sephadex LH-20 (CHCl_3_/MeOH, 2:8, 250 mL) to yield fraction G3-2-1 (80.2 mg). Fraction G3-2-1 was further subjected to CC with CHCl_3_/MeOH (98:2, 150 mL) to give **4** (4.3 mg). Fraction G6 (300.1 mg) was subjected to CC with CHCl_3_/MeOH (9:1, 250 mL) to give two subfractions (G6-1, G6-2). Fraction G6-1 (122.6 mg) was further purified by Sephadex LH-20 (CHCl_3_/MeOH, 2:8, 150 mL) to afford fraction G6-2-1 (68.1 mg). Fraction G6-2-1 was further purified by reverse-phase HPLC (ODS, MeOH/H_2_O, 5:5, 180 mL;) to yield **5 **(7.2 mg). The *n*-butanol soluble portion (80 g) was chromatographed on ODS column eluted with MeOH/H_2_O (0:100, 2,000 mL; 10:90, 2,000 mL; 20:80, 2,000 mL; 40:60, 2,000 mL; 60:40, 2,000 mL; 100:0, 2,000 mL) to give six fractions I, J, K, L, M, N. Fraction J (20 g) was subjected to CC with gradient CHCl_3_/MeOH (100:0, 500 mL; 90:10, 500 mL; 80:20, 500 mL; 60:40, 500 mL; 40:60, 500 mL; 0:100, 500 mL;) to give six fractions (J1–J6). Fraction J1 was further purified by reverse-phase HPLC (ODS, MeOH/H_2_O, 5:95, 150 mL) to yield **1 **(6.9 mg, 2.8 × 10^−^^3^ % of the total extractum 250.0 g).

*Callysponine* (**1**). White amorphous powder; [*α*]^20^_D_ = −52.6 (*c* = 0.41, MeOH); NMR data (CD_3_OD), see Table 1; ESI-MS (*m/z*): 237 [M+Na]^+^, 213 [M-H]^−^; HREIMS (*m/z*): 214.0774 [M]^+ ^(calc. for C_9_H_14_N_2_O_2_S, 214.0776).

*Cyclo-(S-Pro-R-Leu) *(**2**). White crystals; [*α*]^20^_D_ = −78.3 (*c* = 0.03, MeOH).

*Cyclo-(S-Pro-R-Val)* (**3**). White crystals; [*α*]^20^_D_ = −102.6 (*c* = 1.36, MeOH).

*Cyclo-(S-Pro-R-Ala)* (**4**). White solid; [*α*]^20^_D_ = −40.4 (*c* = 0.45, MeOH).

*Cyclo-(S-Pro-R-Tyr)* (**5**). Viscous oil; [*α*]^20^_D_ = −9.3 (*c* = 0.91, MeOH).

## 4. Conclusions

A new 1,4-diazepine, callysponine (**1**), was isolated from South China Sea *Callyspongia *sp. marine sponge, together with four known proline-based diketopiperazines. Callysponine did not show activities against HepG2, A549, and HeLa cell lines.
